# Dynamic control of bacterial antiphage defense through the CdnG–Cap5 cyclic oligonucleotide–based antiphage pathway in *Vibrio cholerae*

**DOI:** 10.1016/j.jbc.2025.111021

**Published:** 2025-12-06

**Authors:** Feng Ye, Jiaao Gong, Yao Ge, Zhao Li, Sen Yin, Ang Gao, Yalan Zhu

**Affiliations:** School of Life Science, Beijing Institute of Technology, Beijing, China

**Keywords:** CdnG, protein structure, second messenger, 3'2'-cGAMP, Cap5, molecular mechanism

## Abstract

The cyclic oligonucleotide–based antiphage signaling system (CBASS) is a key defense mechanism that protects bacteria against viral infections, exhibiting functional homology with the eukaryotic cyclic GMP–AMP synthase–stimulator of interferon gene innate immune pathway. The CBASS immune pathway in *Vibrio cholerae*, a significant human pathogen, positions it as a promising target for novel therapeutic strategies. Here, we report the biochemical and structural characterization of the CdnG–Cap5 CBASS system from *V. cholerae*, a highly abundant and representative clade G system. Our results elucidate the mechanistic basis of CBASS immunity, from second messenger synthesis to effector activation. We demonstrate that *Vc*CdnG produces 3′2′-cyclic GMP–AMP as a bacterial second messenger, which specifically binds the *Vc*Cap5 effector and triggers its tetramerization, leading to cell death. *Vc*Cap5 exhibits multiligand sensitivity and dose-responsive behavior, suggesting a sophisticated strategy for threat-level assessment that allows *V. cholerae* to balance effective antiphage defense. This study provides molecular insights into one of the most widespread CBASS systems and expands our understanding of bacterial immune mechanisms in the ongoing conflict with phages.

The evolutionary arms race between bacteria and bacteriophages has driven the development of sophisticated bacterial immune defense mechanisms against viral (phage) infections (1, 2). Among these, the cyclic oligonucleotide–based antiphage signaling system (CBASS) has emerged as a widespread and evolutionarily conserved antiviral strategy, exhibiting remarkable mechanistic similarities to the eukaryotic cyclic GMP–AMP synthase (cGAS)–stimulator of interferon gene (STING) innate immunity pathway (3, 4, 5, 6). Studies have demonstrated that CBASS is widely distributed in bacterial and archaeal genomes, forming a highly diverse family of antiphage defense systems (3, 4, 7, 8, 9, 10). Moreover, these systems exhibit variability at multiple functional levels, including the oligonucleotide cyclase, the types of second messenger molecules produced, the distinct mechanisms of effector proteins, and ancillary regulatory elements (3, 4, 7, 8, 9, 10). In general, the CBASS system contains two essential components: (1) a cGAS/DncV-like nucleotidyltransferase cyclase (CD-NTase) that functions as a molecular sensor to detect phage infection and subsequently catalyzes the synthesis of cyclic dinucleotide (CDN) or cyclic trinucleotide (CTN) second messengers; and (2) CD-NTase-associated protein (Cap) effectors that specifically recognize and respond to these signaling molecules (4, 7). Upon activation, Cap effectors initiate diverse defense responses, including NAD^+^ degradation, membrane disruption, and nonspecific DNA cleavage, ultimately leading to cell death and containment of phage propagation within bacterial populations (4, 7, 8, 11).

Recent genomic analyses have revealed the remarkable diversity of bacterial CD-NTases, with over 5000 distinct variants classified into eight major clades (A–H) based on sequence homology and structural features (3, 12). To date, only a limited number of CD-NTase structures have been determined, including DncV from *Vibrio cholerae*, CdnB from *Bacteroides fragilis* and *Deinococcus wulumuqiensis*, CdnC from *Escherichia coli*, CdnD from *Enterobacter cloacae*, CdnE from various species, and CdnG from *Bradyrhizobium diazoefficiens* and *Serratia marcescens* (3, 5, 12, 13, 14, 15, 16, 17). While all CD-NTases share a conserved N-terminal nucleotidyltransferase (NTase) domain, their C-terminal helical domain exhibit extensive sequence and structural variability that underpins distinct catalytic properties (3, 18). This architecture of a conserved core coupled with variable regions enables precise substrate selection and strict product specificity. Structural analysis demonstrates that each CD-NTase contains a conserved helix that lines the active-site lid (lid1 and lid2) and positions residues above the acceptor- and donor-nucleotide pockets (12). Lid1, positioned above the acceptor pocket, and lid2, located above the donor pocket, collectively orchestrate the catalytic synthesis of cyclic oligonucleotide signals (12). Bioinformatic analyses have revealed remarkable diversity at these regulatory sites, with 19 amino acid variants at lid1 and 18 amino acids at lid2, representing nearly the full spectrum of natural amino acids (12). Consistent with this structural divergence, biochemical studies have shown that different CD-NTases catalyze the synthesis of chemically diverse nucleotide signals, generating at least 180 distinct cyclic nucleotide species, highlighting their extraordinary capacity for immune signal diversification (8). For example, *V. cholerae* DncV produces 3′3′-cyclic GMP–AMP (cGAMP) to activate the phospholipase CapV, triggering membrane degradation and cell lysis (3, 4, 19); *E. coli* CdnE produce cUA to stimulate the phospholipase CapE, leading to CapE filament formation and membrane disruption (20); *Asticcacaulis* sp. (*As*) CdnG synthesize 3′2′-cGAMP to activate the nuclease activity of Cap5, resulting in genomic DNA degradation (21). Moreover, several CD-NTases have recently been shown to produce CTNs, including 3′3′3′-cAAA by *E. coli* CdnC and 3′3′3′-cAAG by *E. cloacae* CdnD (3, 10, 15). This remarkable diversity of secondary messengers likely enables bacteria to both evade host immune responses and counteract phage-encoded antidefense systems.

Despite the rapid progress in CBASS research in recent years, the mechanistic basis of many CBASS subtypes remains poorly defined because of their extensive diversity and complexity. The CdnG–Cap5 module from *V. cholerae*, a significant human pathogen, constitutes a highly abundant and canonical representative of clade G CBASS systems. In *Vc*CdnG, the lid1 and lid2 residues are asparagine (N) and tyrosine (Y), respectively. This N/Y combination predominates in clade G CBASS systems, reflecting strong evolutionary conservation and functional indispensability. Nevertheless, the molecular mechanism and regulatory features of *V. cholerae* CdnG–Cap5 system have not been systematically characterized. To address this gap, we conducted comprehensive biochemical and structural analyses of the *Vc*CdnG–*Vc*Cap5 defense system. We show that *Vc*CdnG exhibits specific ATP/GTP-binding activity and predominantly synthesizes 3′2′-cGAMP as its major signaling product. Crucially, 3′2′-cGAMP serves as the physiological activator of *Vc*Cap5, triggering *Vc*Cap5 tetramerization and stimulating its dsDNA degradation activity. Furthermore, we demonstrate that *Vc*Cap5 exhibits multiligand sensitivity and dose-dependent activation, suggesting a sophisticated strategy that enables *V. cholerae* to fine-tune antiphage responses according to threat level. Together, these results provide crucial mechanistic insight into clade G CBASS system and expand our understanding of the design principles underlying bacterial innate immunity.

## Results

### *Vc*CdnG synthesizes 3′2′-cGAMP as its primary signaling product

**Figure 1:**
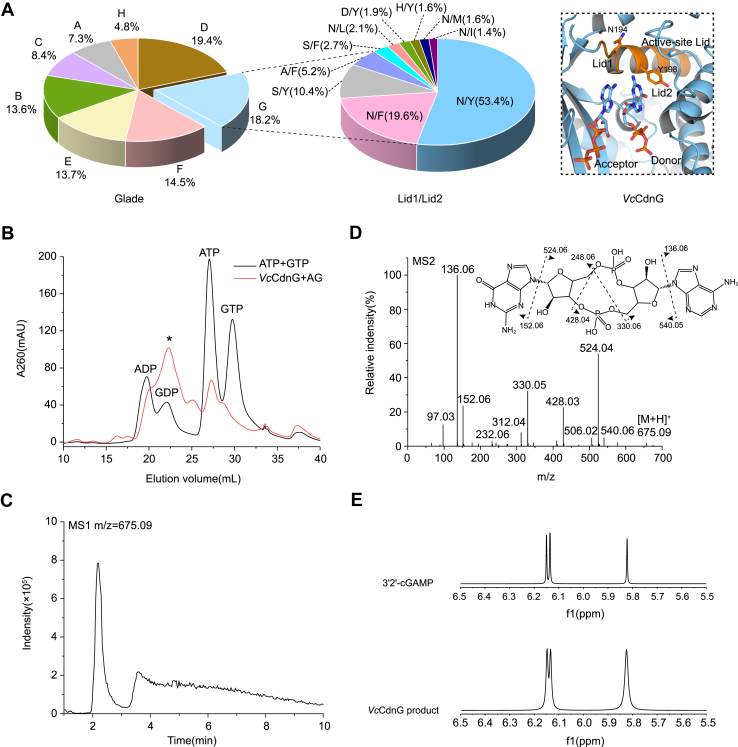
**Biochemical and structural characterization of *Vc*CdnG enzymatic products *in vitro*.***A*, systematic classification of bacterial CD-NTase (*left*), distribution of lid 1 and 2 (*middle*), and close-up view of the *Vc*CdnG catalytic pocket highlighting lid 1 and 2 (*right*). *B*, anion-exchange chromatography analysis of *Vc*CdnG reaction products. The reaction mixture contained 3 μM *Vc*CdnG, 0.2 mM ATP, 0.2 mM GTP, and 10 mM MnCl_2_. A new peak was marked with an *asterisk* (∗). *C*, LC–MS/MS analysis identifies cGAMP as the primary reaction product. Extracted ion chromatogram (EIC) for protonated molecule [M + H]^+^ of cGAMP (*m/z* 675.09). *D*, tandem mass spectrometry (MS/MS) confirms the cyclic dinucleotide. The characteristic fragment ions of the precursor ion at *m/z* 675.09 include the nucleobase ions *m/z* 136.06 (adenine) and *m/z* 152.06 (guanine), the signature cyclic phosphate backbone fragments at *m/z* 540.0 and 524.0 (corresponding to [cGAMP + H - adenine]^+^ and [cGAMP + H – guanine]^+^ or similar structural rearrangements, respectively) and the series of phosphate-containing fragments. *E*, ^1^H NMR spectroscopic validation of the enzymatic product. Stacked ^1^H NMR spectra (600 MHz, D_2_O) of 3′2′-cGAMP standard (*top*) and the purified *Vc*CdnG reaction product (*bottom*). CD-NTase, cGAS/DncV-like nucleotidyltransferase cyclase.

Clade G represents the second largest group of CD-NTases, among which *Vc*CdnG is distinguished by possessing the most widely distributed and characteristic lid motif, a key structural element critical for substrate recognition and catalysis. The lid1 and lid2 residues in *Vc*CdnG are asparagine (N) and tyrosine (Y), respectively. This N/Y combination represents the predominant configuration among clade G CBASS systems, reflecting strong evolutionary conservation and functional indispensability (Fig. 1*A*). To systematically investigate the nucleotide substrate preference and catalytic properties of *Vc*CdnG, we conducted a series of well-controlled biochemical assays. Incubation of purified *Vc*CdnG in optimized reaction buffer containing the essential cofactor Mn^2+^ together with both ATP and GTP produced distinct novel peaks on anion-exchange chromatography, suggesting the synthesis of products (Fig. 1*B*), whereas no new peaks were observed in reaction mixtures lacking Mn^2+^ (Fig. S1*A*).

To determine the chemical identity of the ATP/GTP-derived product, we performed LC–MS/MS. Extended enzymatic reactions with ATP, GTP, and Mn^2+^ yielded a precursor ion peak (*m/z* 675.09), corresponding to the molecular mass of cGAMP (Fig. 1*C*). This assignment was validated by characteristic fragment ion peaks at *m/z* 524.04, 540.06, 330.05, and 428.04 (Fig. 1*D*). NMR spectroscopy further confirmed the linkage type, showing that the chemical shift pattern of the product was consistent with 3′2′-cGAMP (Fig. 1*E*).

We next tested whether *Vc*CdnG can utilize individual ribonucleotide triphosphates as substrates. Reactions containing ATP, GTP, CTP, or UTP were analyzed independently. Distinct cyclic products were detected only in ATP- and GTP-supplemented reactions (Fig. S1, *B* and *C*), whereas no new products were observed in CTP- and UTP-containing systems (Fig. S1*D*). LC–MS/MS revealed that ATP alone yielded c-di-AMP (Fig. S2, *A*–*C*), whereas GTP alone yielded c-di-GMP (Fig. S2, *D*–*F*). These results demonstrate that *Vc*CdnG recognize ATP and GTP as substrates and is capable of synthesizing the corresponding homodimeric CDNs in the absence of the complementary nucleotide.

Collectively, these results indicate that *Vc*CdnG functions as a Mn^2+^-dependent cyclase that selectively synthesizes the bacterial second messenger 3′2′-cGAMP from ATP and GTP. The strict substrate specificity positions *Vc*CdnG as a key enzyme in CBASS-mediated immunity, where 3′2′-cGAMP production likely serves as the central trigger for downstream antiphage defense.

### Overall structure of *Vc*CdnG

**Figure 2:**
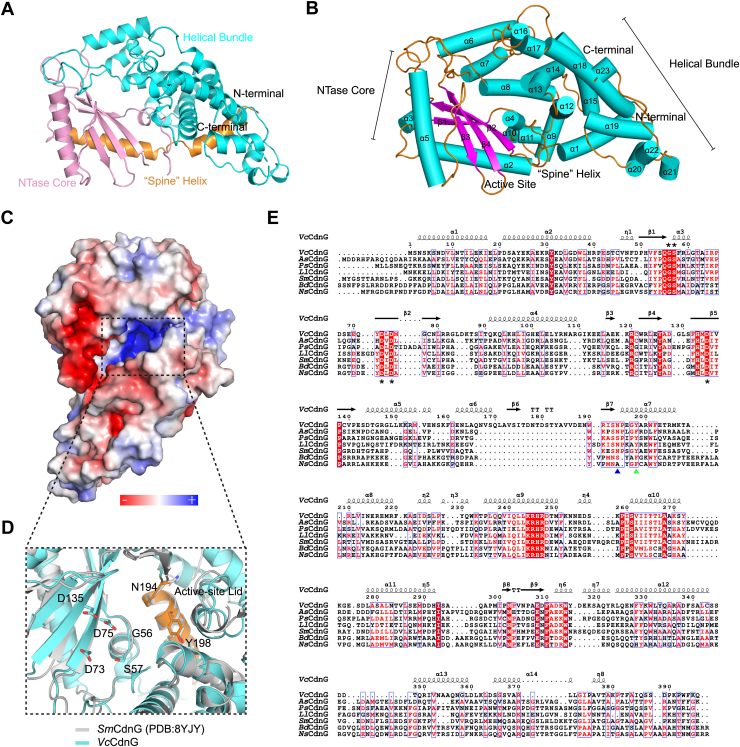
**Overall structure and electrostatic potential surface map of the apo *Vc*CdnG enzyme.***A*, crystallographic structure of apo-*Vc*CdnG. The protein is shown in a *cartoon representation*, with the spine helix in *orange*, the NTase domain in *pink*, and the helical bundle in *cyan*. *B*, secondary structure composition. The same view as in (*A*) with the protein colored by secondary structure: α-helices (*cyan*), β-sheets (*magenta*), and loops/coils (*orange*). *C*, the electrostatic surface potential of the substrate-binding pockets of *Vc*CdnG. A pronounced positive charge cleft (highlighted in *blue*) is observed on the protein surface. *D*, close-up view of the catalytic site. Stick representation of the conserved GS-motif (G56, S57), the metal-coordinating residues (D73, D75, and D135), and the lid1/lid2 residues (N194/Y198) located on the *orange* lid helix. All atoms are colored by element (carbon, *cyan*; nitrogen, *blue*; and oxygen, *red*). *E*, multiple sequence alignment (MSA) of diverse CD-NTases. The conserved GS-motif and catalytic triad are marked with *asterisks* above and below their residues, respectively. The lid1 and lid2 residues are annotated with *blue and green triangles* below them. CD-NTase, cGAS/DncV-like nucleotidyltransferase cyclase.

To elucidate the molecular architecture and catalytic mechanism of the bacterial CDN synthase *Vc*CdnG, we crystallized the full-length protein (residues 1–396) and determined its structure at 2.4 Å resolution by X-ray crystallography (Fig. 2*A* and Table 1). The structure was determined by molecular replacement, employing an AlphaFold 3 (AF3) model as the search template (22).

**Table 1:** X-ray diffraction data collection and refinement statistics

Parameter	*Vc*CdnG (PDB code:9WOO)
Data collection	
Space group	C121
Cell dimensions	
a, b, c (Å)	108.84, 118.11, 78.07
α, β, γ (°)	90.00, 119.49, 90.00
Wavelength (Å)	0.97915
Resolution (Å)	19.95–2.47 (2.56–2.47)
Unique reflections	30,871
Completeness (%)	99.8 (99.9)
Redundancy	3.4
*R*_merge_	0.058 (0.604)
*R*_pim_	0.036 (0.391)
*R*_meas_	0.068 (0.722)
I/σI	14.3 (1.9)
CC_1/2_	0.998 (0.684)
Refinement	
No. of reflections	30,089 (3026)
*R*_work_/*R*_free_	0.197/0.229
No. of atoms	3202
Protein	3141
Ligand/ion	0
Water	61
*B*-factors	69.96
Protein	70.19
Water	57.83
RMSD	
Bond lengths (Å)	0.002
Bond angles (°)	0.42
Ramachandran favored/allowed/outliers (%)	98.18/1.82/0.00
Clashscore	4.33

Values in parentheses are for highest resolution shell.

Consistent with canonical CD-NTases, *Vc*CdnG adopts a characteristic cage-like fold composed of two distinct domains: an N-terminal NTase domain and a C-terminal helical bundle domain (Fig. 2*B*). Specifically, the NTase domain contains a central catalytic core formed by a four-stranded β-sheet (β1–β4) flanked by four α-helices (α2, α3, α4, and α5) (Fig. 2*B*). The C-terminal domain is predominantly composed of 19 α-helices (α1 and α6–α23), forming an extensive helical bundle that likely contributes to the overall structural stability (Fig. 2*B*).

Crucially, the spatial arrangement of these two domains forms a large, solvent-accessible, positively charged catalytic pocket (Fig. 2*C*). This electrostatic feature complements the negatively charged ribonucleotide substrates, thereby facilitating both substrate recognition and product release. Similar to other CD-NTases, detailed structural analysis of this pocket identifies highly conserved active-site residues (D73, D75, and D135) located within the central β-sheet (Fig. 2, *D* and *E*). Adjacent to this sheet, a short α-helix harbors the conserved GS motif (G56 and S57) (Fig. 2, *D* and *E*).

To elucidate the structural differences between *Vc*CdnG and CD-NTases from different clades, we performed structural alignment of *Vc*CdnG with DncV (Protein Data Bank [PDB] code: 4XJ1), CdnB (PDB code: 7LJO), CdnC (PDB code: 6P80), CdnD (PDB code: 7D48), and CdnE (PDB code: 6E0M) (Fig. S3). The analysis reveals that *Vc*CdnG shares a conserved overall architecture with other CD-NTases, all featuring a characteristic N-terminal NTase domain and a C-terminal helical bundle domain. However, *Vc*CdnG exhibits distinct structural features, the α6, α7, and α20 helices, which are absent in several other homologs. Moreover, conformational shifts of approximately ±3 Å were observed in β-strands 2 and 4 within the catalytic center of *Vc*CdnG relative to other clades. These structural divergences suggest that conformational plasticity within the active site may underpin the functional adaptation of CD-NTases to diverse substrate specificities.

Collectively, these findings establish *Vc*CdnG as a representative clade G CD-NTase that employs a conserved catalytic mechanism, while providing the architectural basis for its specific substrate utilization and 3′2′-cGAMP synthesis.

### Structural basis for subtract recognition

**Figure 3:**
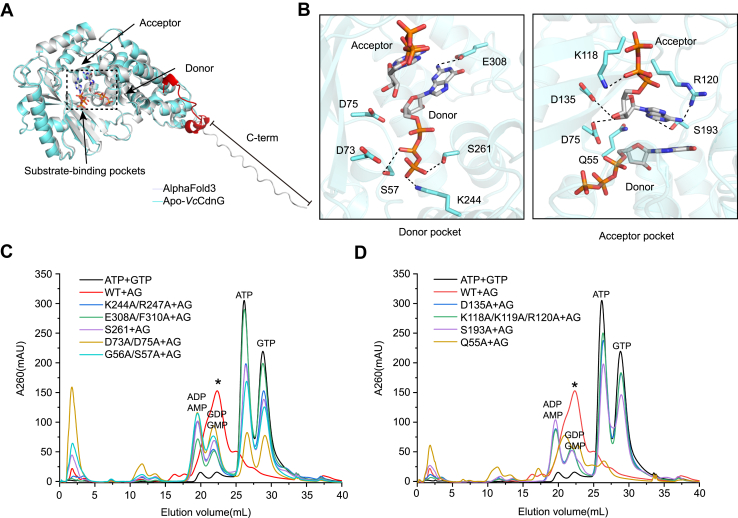
**Molecular mechanism of substrate recognition and catalysis in *Vc*CdnG.***A*, structural validation of the substrate-bound state by AlphaFold3 prediction. Superposition of the experimental apo structure (*cyan*) and the AlphaFold3-predicted holo model (*gray*). Substrates are depicted as *stick models* and colored by element (carbon, *gray*; nitrogen, *blue*; and oxygen, *red*). *B*, mechanistic model of substrate coordination and recognition in *Vc*CdnG. Close-up view of the catalytic pocket highlighting the side chain of key residues. *C* and *D*, functional validation of key residues by alanine mutagenesis. Anion-exchange chromatograms resolve enzymatic reaction products from WT and site-directed mutants targeting the catalytic donor pocket (*C*) and acceptor pocket (*D*). All reactions were performed using 3 μM protein, 0.25 mM ATP, 0.25 mM GTP, and 10 mM MnCl_2_. The new product peak is indicated by an *asterisk* (∗).

Despite extensive efforts, we were unable to experimentally determine the structure of the protein–substrate complex. To gain structural insights into substrate recognition and catalysis, we generated a predicted model of the *Vc*CdnG–ATP/GTP complex using AF3 (Fig. 3*A* and Fig. S4, *A* and *B*). The predicted model closely aligns with the experimentally resolved apo-*Vc*CdnG structure, indicating its reliability and confirming that *Vc*CdnG adopts a stable overall conformation (Fig. 3*A*). The only notable difference lies in the orientation of the C terminus, likely reflecting the inherent flexibility of a disordered region (Fig. 3*A*).

Analysis of the predicted *Vc*CdnG–ATP/GTP complex structure reveals that the donor GTP and acceptor ATP occupy distinct but adjacent binding pockets within the catalytic cavity, forming extensive interactions (Fig. 3*B*). In the donor pocket, the γ-phosphate of the triphosphate group forms hydrogen bonds with polar side chains of K244 and S261, whereas the β-phosphate interacts with S57 (Fig. 3*B*). The guanine base is stabilized by a hydrogen bond with E308, providing nucleotide specificity (Fig. 3*B*). Furthermore, in the acceptor pocket, the β-phosphate of the triphosphate group hydrogen bonds with K118, whereas the ribose group interacts with D75 and D135 (Fig. 3*B*). In addition, the adenosine base is stabilized by hydrogen bonds with R120 and S193 (Fig. 3*B*).

To validate the AF-predicted interactions, we performed *in vitro* enzyme activity assays using a series of *Vc*CdnG mutants. Enzymatic activity was assessed by monitoring both the synthesis of the final CDN product 3′2′-cGAMP and the consumption of the substrates ATP and GTP. Mutations within the donor pocket, including G56A/S57A, K244A/R247A, D73A/D75A, E308A/F310A, and S261A, completely abolished 3′2′-cGAMP production (Fig. 3*C* and Fig. S5, *A*–*D*). Consistently, these mutants showed markedly reduced ATP and GTP utilization, with substrate consumption decreased by approximately 50%, 62%, 26%, 90%, and 57%, respectively, relative to the wildtype (Fig. S6, *A* and *B*). Similarly, mutations in the acceptor pocket (D135A, K118A/K119A/R120A, and S193A) also eliminated 3′2′-cGAMP production, whereas the Q55A retained partial activity, yielding approximately 50% of the wildtype product level (Fig. 3*D* and Fig. S5, *A*–*D*). Correspondingly, substrate utilization by D135A, K118A/K119A/R120A, S193A, and Q55A decreased by ∼74%, ∼68%, ∼46%, and ∼2.8%, respectively (Fig. S6, *A* and *B*). Collectively, these results strongly support the AF-predicted binding mode and confirm the functional importance of these catalytic residues in mediating nucleotide recognition and 3′2′-cGAMP synthesis.

### 3′2′-cGAMP activates the effector protein *Vc*Cap5

**Figure 4:**
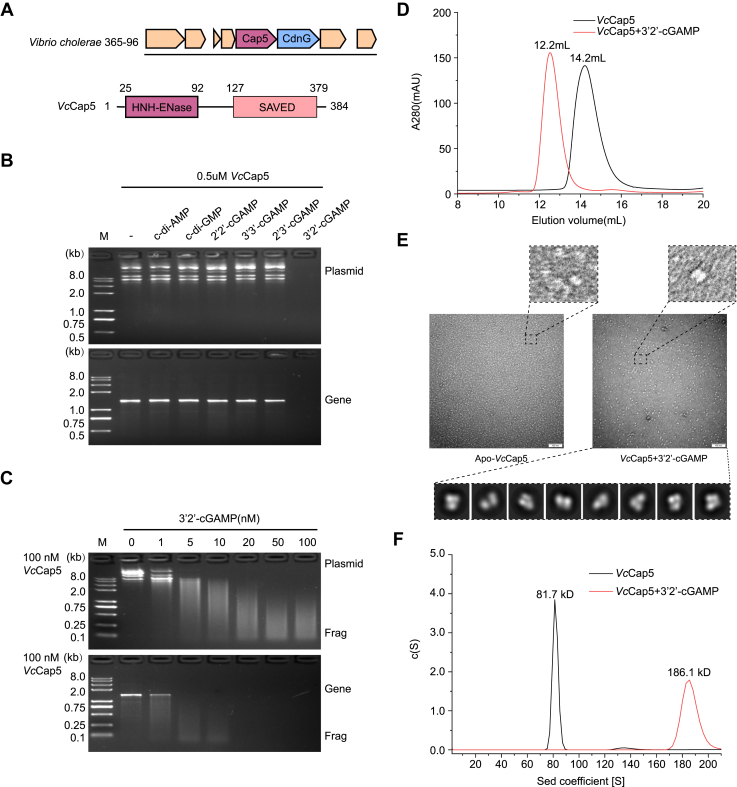
**Biochemical characterization of the DNA cleavage activity of *Vc*Cap5.***A*, schematic view of two operons encoding *Vc*CdnG–Cap5 antiphage defense system (*top*) and domain architecture of *Vc*Cap5 (*bottom*). *B*, agarose gel electrophoresis analysis of DNA degradation assays. Nuclease activity assays were performed by incubating 0.5 μM *Vc*Cap5 with 20 ng/μl DNA in the presence of 0.5 μM of various cyclic dinucleotides. Each gel is a representative of at least three independent experiments. *C*, cleavage assays of supercoiled plasmid (*top*) and linearized DNA (*bottom*) were performed with increasing concentrations of 3′2′-cGAMP. Nuclease activity assays were performed by incubating 0.1 μM *Vc*Cap5 with 20 ng/μl DNA in the presence of 3′2′-cGAMP (1–100 nM). Each gel is a representative of at least three independent experiments. *D*, size-exclusion chromatography (SEC) analysis of apo-*Vc*Cap5 and *Vc*Cap5–3′2′-cGAMP complex. Elution profiles of apo *Vc*Cap5 (*black*) and *Vc*Cap5–3′2′-cGAMP complex (*red*). *E,* electron microscopy visualization of apo-*Vc*Cap5 and *Vc*Cap5–3′2′-cGAMP complex. Representative negative-stain EM micrographs of *Vc*Cap5 in the apo state *(left*) and in the presence of 3′2′-cGAMP *(right*). *F*, sedimentation velocity analysis of apo-*Vc*Cap5 and *Vc*Cap5–3′2′-cGAMP complex.

A gene located downstream of *Vc*CdnG encodes a protein containing an N-terminal HNH endonuclease domain and a C-terminal STING-mediated organellar dynamics and signaling–associated domain and fused to various effector sensor domain (SAVED) (Fig. 4*A*). We propose that this protein, which we named *Vc*Cap5, functions as the cognate effector of *Vc*CdnG. To gain deeper insights into the molecular mechanisms underlying the antiphage defenses mediated by *V. cholerae* CBASS system, we focus on the biochemical characterization of the effector protein *Vc*Cap5.

To characterize the enzymatic activity of *Vc*Cap5, we conducted systematic *in vitro* DNA degradation assays. Six CDNs were tested: 3′2′-cGAMP (the major product of *Vc*CdnG), the minor products c-di-AMP and c-di-GMP, and non-*Vc*CdnG products 2′2′-cGAMP, 3′3′-cGAMP, and 2′3′-cGAMP (Fig. 4*B*). Under conditions of constant protein and DNA substrate concentrations, *Vc*Cap5 exhibited nuclease activity exclusively in the presence of 3′2′-cGAMP (Fig. 4*B*). Notably, its DNA cleavage activity showed a strong dose-dependent response, with progressively enhanced nuclease function at increasing ligand concentrations (Fig. 4*C*). Specifically, at 5 nM 3′2′-cGAMP (with 100 nM *Vc*Cap5), clear substrate cleavage was observed, whereas increasing the ligand concentration to 20 nM resulted in nearly complete digestion of the DNA fragments (Fig. 4*C*).

When the concentration of *Vc*Cap5 was increased, the protein displayed cleavage efficiencies in response to other CDNs. In addition to robust activation by 3′2′-cGAMP, the minor products c-di-GMP and c-di-AMP, as well as the nonproduct 2′2′-cGAMP, were also able to activate *Vc*Cap5 (Fig. S7*A*). Among these, 2′2′-cGAMP exhibited significantly stronger activation capability than c-di-GMP and c-di-AMP (Fig. S7*A*). Specifically, with 1 μM *Vc*Cap5, 1 μM 2′2′-cGAMP produced full cleavage of the substrate DNA, whereas c-di-AMP yielded only partial digestion. At a higher ligand concentration of 5 μM, both 2′2′-cGAMP and c-di-AMP supported complete cleavage, whereas c-di-GMP triggered only partial cleavage (Fig. S7*A*). Consistently, *Vc*Cap5 showed clear dose-dependent cleavage responses to c-di-GMP, c-di-AMP, and 2′2′-cGAMP (Fig. S7*B*). To validate these observations, we performed isothermal titration calorimetry assays to measure the binding affinity between various CDNs and *Vc*Cap5. The results showed that 3′2′-cGAMP binds *Vc*Cap5 with the highest affinity, followed by 2′2′-cGAMP, a trend fully consistent with the nuclease activity assays (Fig. 4*B* and Fig. S7, *A*–*C*).

Together, these findings demonstrate that 3′2′-cGAMP, the primary product of *Vc*CdnG, serves as the primary activator of *Vc*Cap5. Moreover, the *Vc*CdnG–*Vc*Cap5 defense system employs a multiligand activation strategy that enables graded defense responses. This ligand discrimination mechanism provides *V. cholerae* with the capacity to mount proportionate antiphage responses tailored to the repertoire of signaling molecules generated during infection.

### *Vc*Cap5 forms tetramer to execute nuclease activity

To elucidate the activation mechanism of *Vc*Cap5, we performed size-exclusion chromatography (SEC) assays to assess its oligomeric state in the presence of the primary stimulus 3′2′-cGAMP. SEC analysis showed that the elution peak of apo-*Vc*Cap5 appeared at 14.2 ml (Fig. 4*D*). In contrast, incubation with 3′2′-cGAMP resulted in a distinct shift to an earlier elution volume, indicating the formation of a higher molecular weight complex (Fig. 4*D*). Furthermore, negative-stain EM revealed that *Vc*Cap5 particles incubated with 3′2′-cGAMP were markedly larger than those of the apo form (Fig. 4*E*).

To confirm the aggregation state of the *Vc*Cap5–3′2′-cGAMP complex, we performed analytical ultracentrifugation experiments. The results showed that the molecular mass of apo-*Vc*Cap5 was approximately 81.7 kDa (Fig. 4*F*). Given that the theoretical molecular mass of the *Vc*Cap5 monomer is 44.2 kDa, this indicates that apo-*Vc*Cap5 exists as a dimer, consistent with the SEC elution peak at 14.2 ml (Fig. 4*D*). In contrast, the analytical ultracentrifugation results demonstrated that the molecular mass of the *Vc*Cap5–3′2′-cGAMP complex was about 186.1 kDa, approximately twice that of apo-*Vc*Cap5, indicating that 3′2′-cGAMP induces the dimer-to-tetramer transition of *Vc*Cap5 (Fig. 4*F*).

Together, these results demonstrated that 3′2′-cGAMP functions as a stimulator that activates *Vc*Cap5 by inducing its tetramerization, thereby triggering its dsDNA degradation activity.

### Proposed mechanism of 3′2′-cGAMP-activated DNA cleavage by *Vc*Cap5

**Figure 5:**
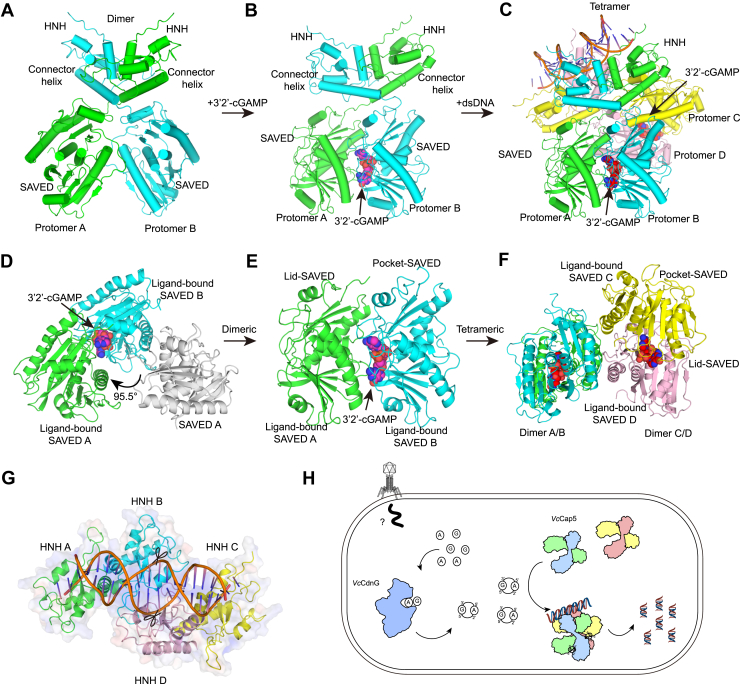
**Proposed model for oligomerization-activated DNA cleavage by *Vc*Cap5.***A*, dimer structure of *Vc*Cap5 predicted by AlphaFold3. *B*, structural features of ligand-bound *Vc*Cap5 dimers. *C*, DNA-bound tetramer structure predicted by AlphaFold3. *D*, ligand-induced structural transition in the SAVED domain of *Vc*Cap5. *E*, structural features of ligand bound in the SAVED domain of *Vc*Cap5. *F*, ligand-induced dimer-to-tetramer transition in the SAVED domain of *Vc*Cap5. *G*, a schematic representation of the tetramer in the HNH domain of *Vc*Cap5 cleaving the *orange* dsDNA. *H*, mechanistic model of *Vc*CdnG–*Vc*Cap5-mediated antiphage defense. The four subunits A, B, C, and D are colored in *green*, *blue*, *yellow*, and *pink*, respectively, and rendered in *cartoon representation*. SAVED A is colored *gray* to represent the absence of ligand. The HNH and SAVED domains are indicated. Ligand 3′2′-cGAMP is shown as a *sphere*. Scissors are used to denote the location of DNA cleavage by the HNH domain. SAVED, STING-mediated organellar dynamics and signaling–associated domain and fused to various effector sensor domain.

Previous studies have shown that Cap5 proteins from *Pseudomonas syringae* (*Ps*), *Lactococcus lactis* (*Ll*), and *As* sp. share a conserved architecture comprising an N-terminal HNH endonuclease domain and a C-terminal SAVED domain. Activation through binding of the second messenger 3′2′-cGAMP triggers a dimer-to-tetramer transition that properly positions the HNH domains for DNA cleavage (21, 23, 24). To gain structural insight into *Vc*Cap5 oligomeric transition, we predicted its dimeric and tetrameric states using AF3 (Fig. 5, *A* and *C* and Fig. S8, *A* and *B*). The predicted dimer adopts a crisscross arrangement with protomers A and B related by twofold symmetry (Fig. 5*A*), whereas the tetramer consists of two such dimers (A/B and C/D) forming a similar crisscross configuration—an architecture consistent with those reported for *Ps*Cap5, *Ll*Cap5, and *As*Cap5 (21, 23, 24) (Fig. 5*C*).

Structural analysis of the predicted tetrameric *Vc*Cap5 revealed that the primary ligand-binding pocket is formed mainly by the SAVED domain of protomer B (or D), whereas the SAVED domain of protomer A (or C) forming a lid over this pocket (Fig. 5, *B*, *E* and *F*). Consequently, the two protomers within each A/B dimer (or C/D) engage in asymmetric interactions with the ligand (Fig. 5, *B*, *E*, and *F*). Furthermore, ligand binding induces closure and rotational movement of the SAVED domains relative to both the HNH endonuclease domains and the connector helices (Fig. 5, *B* and *C*). Structural comparison with the apo state (dimeric state) demonstrates that the SAVED domain of protomer A undergoes a rotation of ∼95.5°, thereby reorienting its opposite surface toward the SAVED domain of protomer B (Fig. 5*D*). In the activated tetrameric state, the HNH domains of protomers B and D are positioned adjacent to each other in catalytically active states, enabling DNA cleavage capability (Fig. 5*G*). In contrast, the HNH domains of protomers A and C reside at the periphery of the tetrameric assembly in catalytically incompetent states (Fig. 5*G*).

Structural superposition of the predicted *Vc*Cap5 tetramer with *Ps*Cap5 revealed severe steric clashes in the global alignment (RMSD = 20.388 Å), whereas the local alignments of individual subunits closely match the corresponding regions of the *Ps*Cap5 (Fig. S7*D*). Specifically, when superposition is performed based on protomers A/B or C/D, the predicted protomer A/B or C/D closely matches its counterpart in the *Ps*Cap5 tetramer, with RMSD values of 2.712 Å and 2.701 Å, respectively (Fig. S7*D*). These observations suggest that CdnG–Cap5 system share a conserved activation paradigm in different species but exhibit distinct conformational rearrangements during activation.

Based on the biochemical and structural data, we propose a mechanistic model for ligand-activated DNA degradation by *Vc*Cap5. In the apo state, the *Vc*Cap5 homodimer is inactive. Binding of 3′2′-cGAMP to the dimer induces an open-to-closed transition of the SAVED domains, creating an interface conducive to tetramer formation. This conformational rearrangement propagates to the HNH endonuclease domains, activating two of them catalytically and positioning them in close proximity to facilitate DNA degradation (Fig. 5).

## Discussion

In this study, we elucidated the molecular mechanism of the *Vc*CdnG–*Vc*Cap5 defense system, a clade G CBASS system in *V. cholerae*, through biochemical and structural biological approaches. We demonstrated that *Vc*CdnG functions as a Mn^2+^-dependent cyclase that selectively utilizes ATP and GTP to synthesize 3′2′-cGAMP and showed for the first time that this molecule serves as the activator of the effector protein *Vc*Cap5, inducing tetramerization to trigger dsDNA degradation. Based on these findings, we propose a model to explain the antiphage immune mechanism of CBASS from *V. cholerae* (Fig. 5*H*). These findings not only reveal the unique activation mechanism of the clade G CBASS system but also provide new insights into how bacteria achieve precise immunity through second messenger diversity.

Furthermore, we confirmed that *Vc*CdnG preferentially uses ATP and GTP to synthesize 3′2′-cGAMP and can generate c-di-AMP or c-di-GMP in the presence of a single nucleotide. This catalytic plasticity may represent an evolutionary adaptation, enabling the host to respond to diverse phage infections. Moreover, the SAVED domain of *Vc*Cap5 senses multiple cyclic oligonucleotides (3′2′-cGAMP, c-di-GMP, c-di-AMP, and 2′2′-cGAMP) but exhibits the highest affinity for 3′2′-cGAMP, the primary product of *Vc*CdnG, with activation efficiency showing a graded pattern. This suggests that *V. cholerae* may differentiate the threat level of phage infection based on structural differences in CDN molecules: when high-affinity ligands are present, the HNH nuclease is strongly activated for rapid clearance; whereas low-affinity ligands may elicit a moderate response, thus preventing unnecessary cell death.

Biochemical analyses demonstrate that binding of 3′2′-cGAMP to *Vc*Cap5 induces its dimer-to-tetramer transition. This tetramer-dependent activation mechanism is consistent with observations in other CBASS effectors (23, 24), underscoring its evolutionary conservation across bacterial immune systems. Furthermore, the multiligand sensing strategy of *Vc*Cap5 and its graded responsiveness to different CDNs represent a sophisticated regulatory mechanism that ensures precise modulation of antibacterial activity. This system enables *V. cholerae* to initiate immune responses tailored to the signaling profiles of infecting phages, thereby maintaining equilibrium between effective defense and cellular death.

Our study establishes the core activation mechanism of the *V. cholerae* CdnG–Cap5 system, and a comparative analysis with previously characterized homologs reveals both conserved features and key distinctions. Similar to the *Ps*, *Ll*, and *As* sp. CdnG–Cap5 systems, whose CdnG enzymes also produce 3′2′-cGAMP to activate their respective Cap5 effector, *Vc*Cap5 employs a conserved SAVED domain for ligand sensing and undergoes 3′2′-cGAMP–induced tetramerization to activate its HNH nuclease domain (21, 23, 24). However, the *V. cholerae* system exhibits unique functional specialization in several aspects. First, *Vc*CdnG demonstrates distinct catalytic plasticity, while it preferentially synthesizes 3′2′-cGAMP from ATP/GTP, it retains the capacity to produce homodimeric CDNs (c-di-AMP and c-di-GMP) under substrate-limited conditions, a feature not observed in other well-characterized clade G synthases. Second, *Vc*Cap5 displays a broader ligand sensitivity profile, responding not only to its cognate signal 3′2′-cGAMP but also to c-di-GMP, c-di-AMP, and 2′2′-cGAMP with graded efficiency. These comparative insights underscore the evolutionary conservation and diversification within clade G CBASS systems.

The elucidation of the CBASS immune pathway in *V. cholerae*, a significant human pathogen, positions it as a promising target for novel therapeutic strategies. Our mechanistic insights into CBASS activation from *V. cholerae* enable the rational design of “CBASS-evading” or “CBASS-suppressing” phage variants. One promising approach involves engineering phage to express enzymes that degrade the secondary messenger 3′2′-cGAMP, effectively disarming this bacterial defense system prior to its activation. Such targeted interventions could mitigate the broad selective pressures that drive conventional antibiotic resistance, offering a more sustainable antimicrobial paradigm.

In summary, the *Vc*CdnG–*Vc*Cap5 CBASS system employs a dual strategy of catalytic versatility and effector sensitivity to confer pathogen-specific immunity. This refined mechanism deepens our understanding of bacterial innate immunity and highlights the role of second messenger diversity in adaptive defense strategies. Beyond their biological significance, these insights may inform the development of novel antimicrobials targeting nucleotide signaling pathways in pathogenic bacteria.

## Experimental procedures

### Bacterial strains and cultures

All experiments utilized *E. coli* strains. *E. coli* TOP10 (Tsingke) was used for plasmid cloning and propagation, cultured at 37 °C in LB medium supplemented with 50 μg/ml kanamycin. *E. coli* BL21 (DE3) (Tsingke) was employed for recombinant protein expression. Synthetic CDNs, including c-di-GMP, c-di-AMP, 3′3′-cGAMP, 2′3′-cGAMP, 2′2′-cGAMP (MedChemExpress), and 3′2′-cGAMP (BioLoG) were used for biochemical assays.

### Plasmid construction and point mutants

The *Vc*CdnG gene from *V. cholerae* 365-96 (IMG gene accession: 2633935099) was synthesized and codon-optimized for expression in *E. coli* by the Sangon Biotech. The optimized gene was cloned into the multiple cloning site 1 (BamHI/NotI) of the pRSF-Duet-1 vector. A 6× His-SUMO tag followed by a ubiquitin-like protease 1 (ULP1) cleavage site was incorporated at the N terminus of the insertion to facilitate subsequent protein purification. Positive clones were screened by colony PCR, and plasmid DNA was extracted using commercial kit. Insert sequences were verified by Sanger sequencing. All point mutations were generated using PCR-based site-directed mutagenesis with the wildtype plasmid as template. The corresponding *Vc*Cap5 genes (IMG gene accession: 2633935098) were constructed using the same procedure as for *Vc*CdnG genes.

### Protein expression and purification

Recombinant proteins were overexpressed in *E. coli* BL21 (DE3) strain in LB medium at 37 °C with shaking at 220 rpm until the absorbance at 600 nm reached 0.8. After cooling to 20 °C, expression was induced with 0.5 mM IPTG and continued overnight at the same temperature. Cells were harvested, resuspended in binding buffer (50 mM Tris–HCl, 500 mM NaCl, 20 mM imidazole, 0.035% β-mercaptoethanol, 5% glycerol, and 1 mM DTT) and lysed at 4 °C using high-pressure cracker at 1200 bar. The lysate was centrifuged at 18,000 rpm for 30 min to remove debris. The supernatant was applied on 10 ml Ni^2+^–NTA affinity column pre-equilibrated with binding buffer. Target proteins were eluted with elution buffer containing 50 mM Tris–HCl (pH 7.5), 500 mM NaCl, 300 mM imidazole, 5% glycerol, 0.035% β-mercaptoethanol, and 1 mM DTT.

The eluted protein was incubated with ULP1 protease for 8 h at 4 °C to cleave 6× His-SUMO tag, followed by overnight dialysis with imidazole-free buffer. The cleaved sample was then applied to a 5 ml Ni^2+^–NTA column to remove uncleaved protein, the His-SUMO tag, and ULP1. The flow-through was collected, concentrated, and further purified by SEC on a Superdex 200 16/60 column pre-equilibrated with buffer B (20 mM Tris [pH 7.5], 200 mM NaCl, and 1 mM DTT). Target protein peaks were concentrated *via* 10 kDa ultracentrifugation filter, analyzed by SDS-PAGE with Coomassie staining, and stored at −80 °C. All mutant proteins were purified as described for wildtype proteins.

### Synthesis and analysis of *Vc*CdnG products

CTN synthesis was performed essentially as described (3, 10). Recombinant *Vc*CdnG (3 μM) was incubated overnight at 37 °C in a reaction mixture (1 ml) containing 0.25 mM ATP, 0.25 mM GTP, 10 mM Tris–HCl (pH 7.5), 10 mM NaCl, 10 mM MnCl_2_, and 1 mM DTT. The reaction was terminated by heating at 95 °C for 5 min. After centrifugation at 13,000 rpm for 15 min to remove precipitated protein, the supernatant was subjected to ion-exchange chromatography on a Resource Q column (1 ml) with a 0 to 2 M ammonium acetate gradient. Product peaks were pooled, evaporated under vacuum, and resuspended in water for further analysis.

### Product identification by LC–MS/MS

Reaction products were analyzed using an Agilent 6530 Q-TOF mass spectrometer coupled with an MS/MS system operating in positive electrospray ionization mode (3). Separation was performed on a Kinetex C18 column (2.1 × 100 mm, 2.6 μm) maintained at 25 °C, with a flow rate of 0.2 ml/min. The mobile phase consisted of solvent A (10 mM ammonium acetate, pH 5.0) and solvent B (acetonitrile), using the following gradient: 1% B at 0 min, increased to 70% B by 13 min, held until 17 min, and returned to 1% B by 20 min. *Vc*CdnG products were identified by targeted exact mass analysis using MassHunter Qualitative Analysis software (version B.06.00; Agilent).

### Chemical bond characterization *via*^1^H NMR spectroscopy

CDNs were dissolved in 500 μl of D_2_O and analyzed on a 600 MHz Agilent DD2 spectrometer at 25 °C. Commercial 3′2′-cGAMP was used as a control and processed under identical conditions. All spectra were processed and visualized using MestReNova software (Mestrelab Research S.L.).

### DNA degradation assays

DNA cleavage assays were performed as previously described with modifications (23, 24). Briefly, *Vc*Cap5 (0.5 μM) was preincubated with 0.1 μM commercial CDNs on ice for 30 min in 20 μl reaction buffer (10 mM Tris–HCl [pH 7.5], 25 mM NaCl, 5 mM MgCl_2_, and 1 mM DTT). The reaction was initiated by adding 0.4 μg of pRSF-Duet plasmid or PCR product, followed by incubation at 37 °C for 20 min. Reactions were terminated with 5 μl of 25% glycerol. Samples were resolved on a 1.5% agarose gel containing 1 μg/ml ethidium bromide at 120 V for 30 min and visualized using an iBright CL1500 Imaging System.

### Size-exclusion chromatography

Purified *Vc*Cap5 were diluted to 3 mg/ml in buffer containing 20 mM Tris–HCl (pH 7.5), 200 mM NaCl, and 1 mm DTT. Samples were incubated on ice for 30 min either alone or with 3′2′-cGAMP (at 1.2-fold molar excess relative to protein), followed by centrifugation at 13,000 rpm for 10 min at 4 °C. The supernatant was then injected onto a Superdex 200 size-exclusion column.

### Isothermal titration calorimetry

A total of 19 successive injections of 2 μl 150 μM 3′2′-cGAMP were titrated into the sample cell containing 15 μM *Vc*Cap5 at 25 °C, with 120 s intervals between injections. Data were processed using Origin software (OriginLab Corporation), including baseline correction, peak integration, and fitting to a two-site binding model.

### Analytical ultracentrifugation

For sedimentation velocity experiments, *Vc*Cap5 was diluted to a final concentration of 0.8 mg/ml in buffer containing 20 mM Tris–HCl (pH 7.5) and 200 mM NaCl. Samples were loaded into a two-channel charcoal-filled Epon centerpiece and sealed with quartz windows. Sedimentation was performed in an Optima AUC (Beckman Coulter) equipped with both UV absorbance and interference optical systems. Data were acquired at 55,000 rpm and 20 °C. Continuous (c[s]) distribution models were fitted to the data using SEDFIT software (National Institutes of Health) (25). The partial specific volume of the protein, buffer density, and viscosity were calculated using SEDNTERP (John S. Philo, Alliance Protein Laboratories) (26).

### Negative-stain EM

Protein samples (0.03 mg/ml, 4 μl) were applied to glow-discharged continuous carbon grids, followed by staining with two drops of 2% uranyl formate. After air-drying, grids were imaged using a Tecnai Spirit electron microscope (FEI) operated at 100 kV. Images were acquired at a nominal magnification of 120,000× with a CCD camera.

### Crystallization and structure determination

*Vc*CdnG was concentrated to 10 mg/ml and crystallized at 16 °C using the sitting-drop vapor diffusion method with a reservoir solution containing 0.1 M Hepes (pH 7.5) and 4.3 M sodium chloride. Crystals were cryoprotected in reservoir solution supplemented with 20% glycerol and flash-cooled in liquid nitrogen. Diffraction data were collected at beamline BL18U1 of the Shanghai Synchrotron Radiation Facility and processed with the HKL package. Phases were determined by molecular replacement in PHENIX (Lawrence Berkeley National Laboratory) using Phaser with an AF3-predicted model as the search probe (27, 28). Iterative model building and refinement were performed in Coot (29, 30) and PHENIX (28), respectively. Data collection and refinement statistics are summarized in Table 1. All molecular graphic figures were prepared using PyMOL, version 3.1.6.1 (Schrödinger LLC) (31).

## Data availability

The data that support this study are available from the corresponding authors upon request. The coordinates have been deposited into the PDB under accession number 9WOO.

## Supporting information

This article contains supporting information.

## Conflict of interest

The authors declare that they have no conflicts of interest with the contents of this article.

## References

[1] Bernheim A., Sorek R. (2020). The pan-immune system of bacteria: antiviral defence as a community resource. Nat. Rev. Microbiol..

[2] Georjon H., Bernheim A. (2023). The highly diverse antiphage defence systems of bacteria. Nat. Rev. Microbiol..

[3] Whiteley A.T., Eaglesham J.B., de Oliveira Mann C.C., Morehouse B.R., Lowey B., Nieminen E.A. (2019). Bacterial cGAS-like enzymes synthesize diverse nucleotide signals. Nature.

[4] Cohen D., Melamed S., Millman A., Shulman G., Oppenheimer-Shaanan Y., Kacen A. (2019). Cyclic GMP-AMP signalling protects bacteria against viral infection. Nature.

[5] Morehouse B.R., Govande A.A., Millman A., Keszei A.F.A., Lowey B., Ofir G. (2020). STING cyclic dinucleotide sensing originated in bacteria. Nature.

[6] Patel D.J., Yu Y., Jia N. (2022). Bacterial origins of cyclic nucleotide-activated antiviral immune signaling. Mol. Cell.

[7] Millman A., Melamed S., Amitai G., Sorek R. (2020). Diversity and classification of cyclic-oligonucleotide-based anti-phage signalling systems. Nat. Microbiol..

[8] Slavik K.M., Kranzusch P.J. (2023). CBASS to cGAS-STING: the origins and mechanisms of nucleotide second messenger immune signaling. Annu. Rev. Virol..

[9] Jenson J.M., Chen Z.J. (2024). cGAS goes viral: a conserved immune defense system from bacteria to humans. Mol. Cell.

[10] Ye Q., Lau R.K., Mathews I.T., Birkholz E.A., Watrous J.D., Azimi C.S. (2020). HORMA domain proteins and a Trip13-like ATPase regulate bacterial cGAS-like enzymes to mediate bacteriophage immunity. Mol. Cell.

[11] Duncan-Lowey B., Kranzusch P.J. (2022). CBASS phage defense and evolution of antiviral nucleotide signaling. Curr. Opin. Immunol..

[12] Govande A.A., Duncan-Lowey B., Eaglesham J.B., Whiteley A.T., Kranzusch P.J. (2021). Molecular basis of CD-NTase nucleotide selection in CBASS anti-phage defense. Cell Rep..

[13] Kato K., Ishii R., Hirano S., Ishitani R., Nureki O. (2015). Structural basis for the catalytic mechanism of DncV, bacterial homolog of cyclic GMP-AMP synthase. Structure.

[14] Yang C.S., Shie M.Y., Huang S.W., Wang Y.C., Hou M.H., Chen C.J. (2025). Structural insights into signaling promiscuity of the CBASS anti-phage defense system from a radiation-resistant bacterium. Int. J. Biol. Macromol..

[15] Ko T.P., Wang Y.C., Tsai C.L., Yang C.S., Hou M.H., Chen Y. (2021). Crystal structure and functional implication of a bacterial cyclic AMP-AMP-GMP synthetase. Nucleic Acids Res..

[16] Yang C.S., Ko T.P., Chen C.J., Hou M.H., Wang Y.C., Chen Y. (2023). Crystal structure and functional implications of cyclic di-pyrimidine-synthesizing cGAS/DncV-like nucleotidyltransferases. Nat. Commun..

[17] Yan Y., Xiao J., Huang F., Xian W., Yu B., Cheng R. (2024). Phage defence system CBASS is regulated by a prokaryotic E2 enzyme that imitates the ubiquitin pathway. Nat. Microbiol..

[18] Kranzusch P.J. (2019). cGAS and CD-NTase enzymes: structure, mechanism, and evolution. Curr. Opin. Struct. Biol..

[19] Severin G.B., Ramliden M.S., Hawver L.A., Wang K., Pell M.E., Kieninger A.K. (2018). Direct activation of a phospholipase by cyclic GMP-AMP in El Tor Vibrio cholerae. Proc. Natl. Acad. Sci. U. S. A..

[20] Wang J., Li Z., Lang H., Fu W., Gao Y., Yin S. (2025). Cyclic-dinucleotide-induced filamentous assembly of phospholipases governs broad CBASS immunity. Cell.

[21] Fatma S., Chakravarti A., Zeng X., Huang R.H. (2021). Molecular mechanisms of the CdnG-Cap5 antiphage defense system employing 3',2'-cGAMP as the second messenger. Nat. Commun..

[22] Jumper J., Evans R., Pritzel A., Green T., Figurnov M., Ronneberger O. (2021). Highly accurate protein structure prediction with AlphaFold. Nature.

[23] Rechkoblit O., Sciaky D., Ni M., Li Y., Kottur J., Fang G. (2025). Mechanism of DNA degradation by CBASS Cap5 endonuclease immune effector. Nat. Commun..

[24] Rechkoblit O., Sciaky D., Kreitler D.F., Buku A., Kottur J., Aggarwal A.K. (2024). Activation of CBASS Cap5 endonuclease immune effector by cyclic nucleotides. Nat. Struct. Mol. Biol..

[25] Schuck P. (2000). Size-distribution analysis of macromolecules by sedimentation velocity ultracentrifugation and lamm equation modeling. Biophys. J..

[26] Vistica J., Dam J., Balbo A., Yikilmaz E., Mariuzza R.A., Rouault T.A. (2004). Sedimentation equilibrium analysis of protein interactions with global implicit mass conservation constraints and systematic noise decomposition. Anal. Biochem..

[27] Liebschner D., Afonine P.V., Baker M.L., Bunkóczi G., Chen V.B., Croll T.I. (2019). Macromolecular structure determination using X-rays, neutrons and electrons: recent developments in Phenix. Acta Crystallogr. D Struct. Biol..

[28] Adams P.D., Afonine P.V., Bunkóczi G., Chen V.B., Davis I.W., Echols N. (2010). PHENIX: a comprehensive Python-based system for macromolecular structure solution. Acta Crystallogr. D Biol. Crystallogr..

[29] Emsley P., Lohkamp B., Scott W.G., Cowtan K. (2010). Features and development of Coot. Acta Crystallogr. D Biol. Crystallogr..

[30] Casañal A., Lohkamp B., Emsley P. (2020). Current developments in Coot for macromolecular model building of Electron Cryo-microscopy and Crystallographic Data. Protein Sci..

[31] Schrödinger L. (2025).

