# Supplementary material for: Dynamic control of bacterial antiphage defense through the CdnG–Cap5 cyclic oligonucleotide–based antiphage pathway in Vibrio cholerae
Source: J Biol Chem. 2025 Dec 6;302(1):111021. doi: 10.1016/j.jbc.2025.111021 (PMC12804361; doi:10.1016/j.jbc.2025.111021)
Supplement: Supporting Information [file mmc1.pdf]

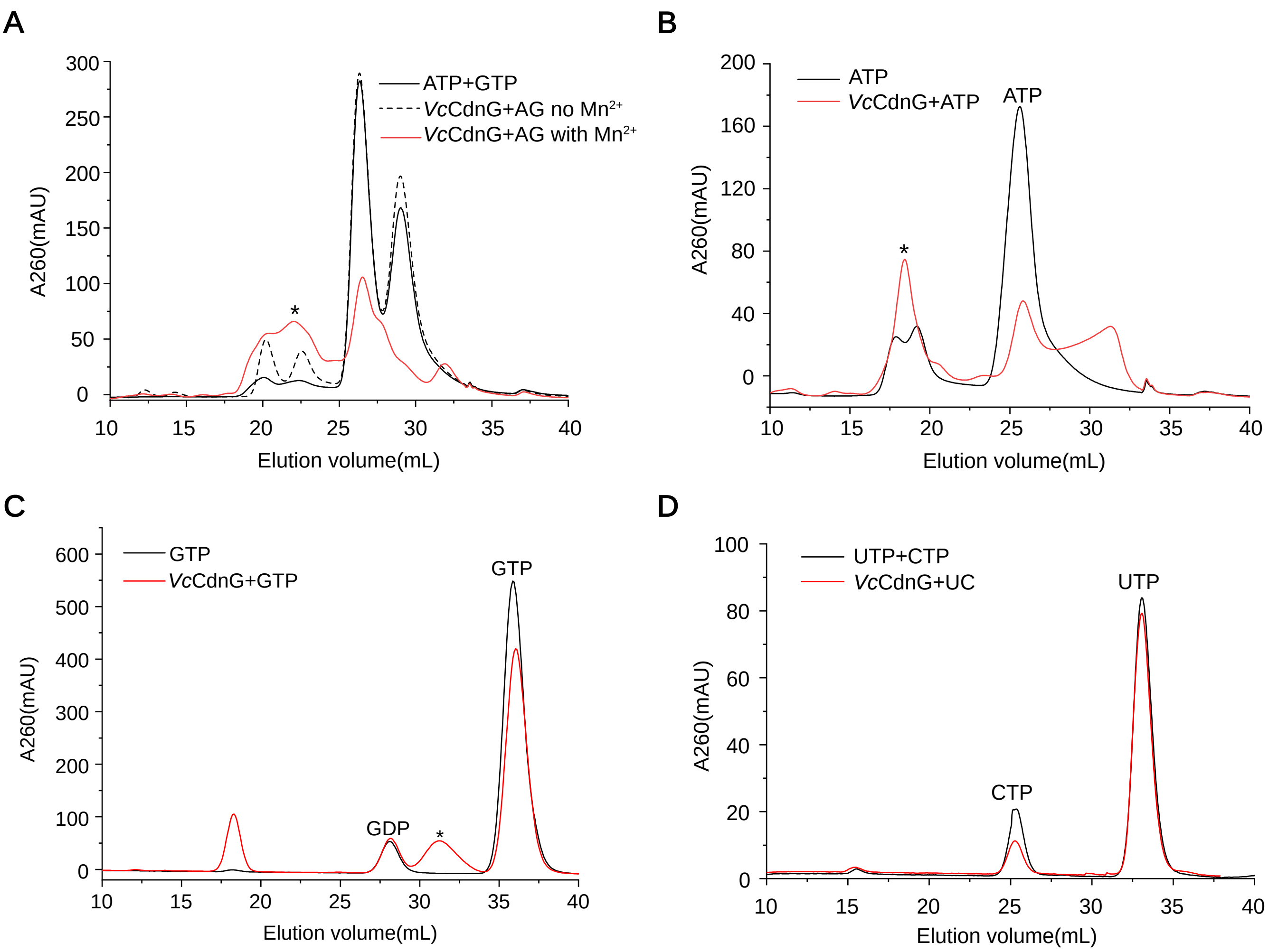

Figure S1. Anion-exchange chromatography of *Vc*CdnG reaction products with NTPs. *A*, chromatographic analysis of the Mn^{2+}-dependent enzymatic reaction. *B*-*D*, chromatographic analysis of the enzymatic reaction utilizing ATP (*A*), GTP (*B*) or UTP and CTP (*C*) as a substrate. The asterisks in panels *A, B*, and *C* of the figure denote the enzymatic products 3'2'-cGAMP, c-di-AMP, and c-di-GMP, respectively.

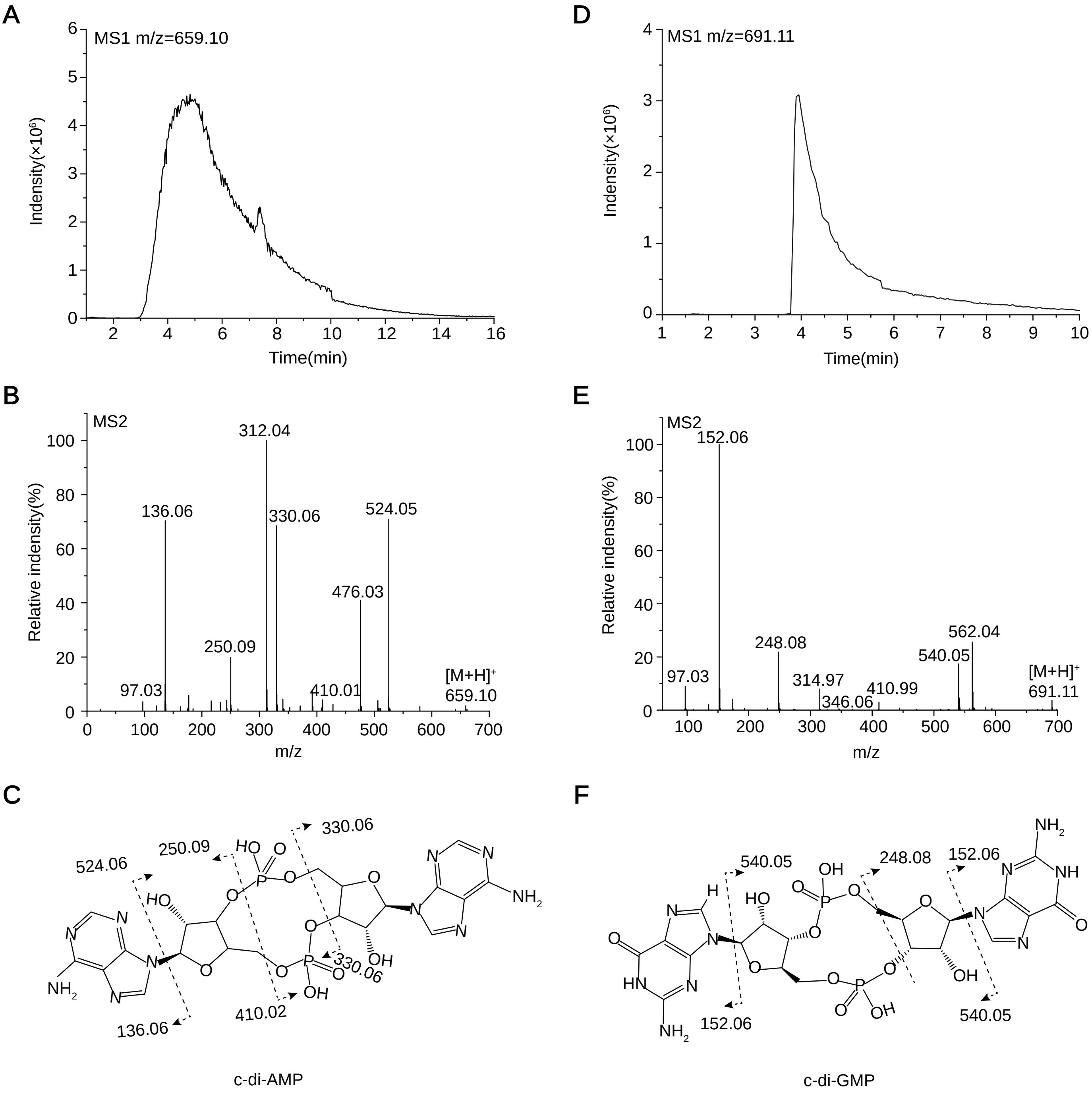

Figure S2. Identification of minor product in the *Vc*CdnG catalytic reaction. *A*, *D*, extracted ion chromatogram (EIC) for protonated molecule [M+H]+ of c-di-GMP (*m/z* 691.11) (*A*) and protonated molecule [M+H]+ of c-di-AMP (*m/z* 659.10) (*D*). *B*, the characteristic fragments include *m/z* 152.06 (protonated guanine) and *m/z* 540.05 ([c-di-GMP + H - guanine]$^+$). *C*, *F*, chemical structure of c-di-GMP (*C*) and c-di-AMP (*F*). *E*, the characteristic fragment ions include the nucleobase ions *m/z* 136.06 (protonated adenine) and *m/z* 524.05 ([c-di-AMP + H - adenine]$^+$).

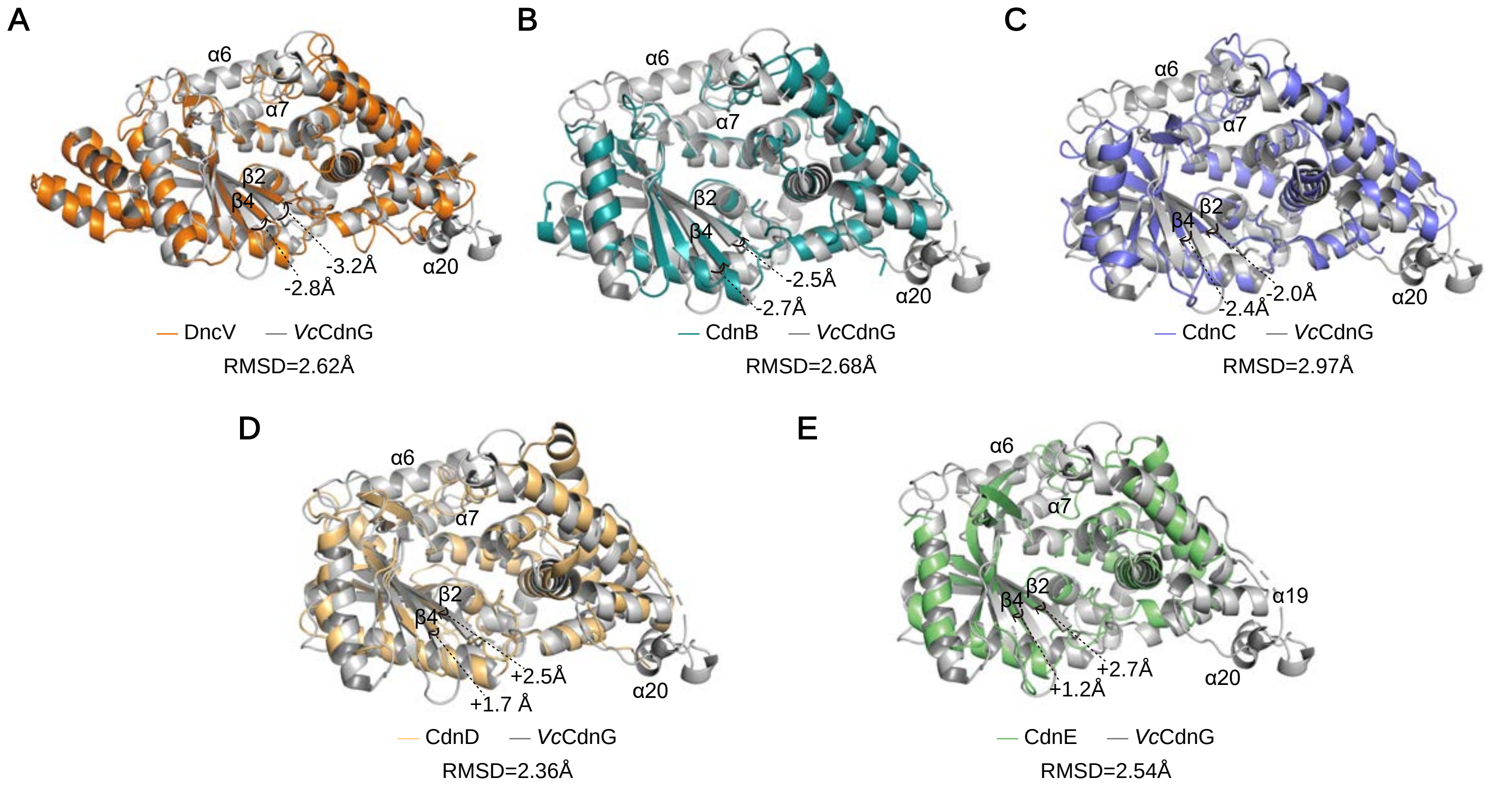

Figure S3. Structural comparison of *Vc*CdnG with various CD-NTases. Structural superpositions of *Vc*CdnG with clade A (DncV, PDB: 4XJ1), clade B (CdnB, PDB: 7LJO), clade C (CdnC, PDB: 6P80), clade D (CdnD, PDB: 7D48), and clade E (CdnE, PDB: 6E0M) are shown in panels *A*-*E*, respectively. In all panels, *Vc*CdnG is colored in gray, and the comparative CD-NTases are colored as follows: *orange*, *deepteal*, *slate*, *lightorange*, and *lime*. Each structural superposition panel displays the corresponding Cα RMSD value beneath it. The conformational shift in the β-sheet containing the catalytic triad, identified by structural alignment, is indicated in the figure, where the '+' and '-' signs denote movements away from and toward the substrate, respectively.

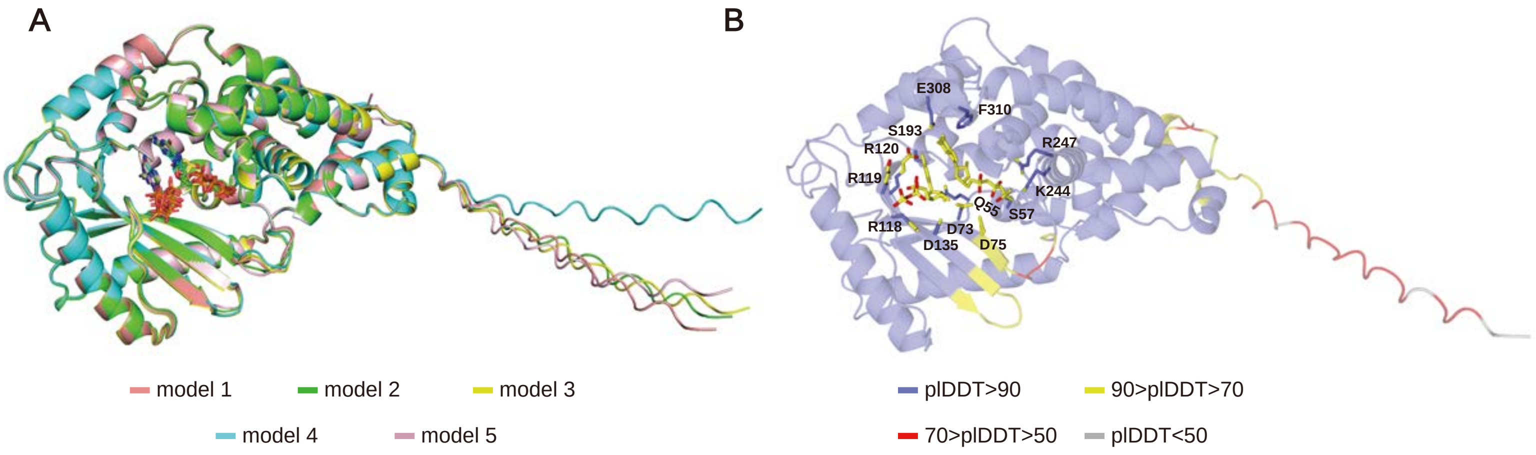

Figure S4. The *Vc*CdnG–ATP–GTP ternary complex structure computed by AlphaFold. *A*, superposition of the five best *Vc*CdnG–ATP–GTP ternary complex models. ATP and GTP are depicted as sticks. *B*, predicted structure of the *Vc*CdnG–ATP–GTP complex colored by pLDDT: *slate* (plDDT > 90), *yellow* (90 > plDDT > 70), *red* (70 > plDDT > 50), and *gray* (plDDT < 50). Mutation sites, ATP and GTP are shown as sticks.

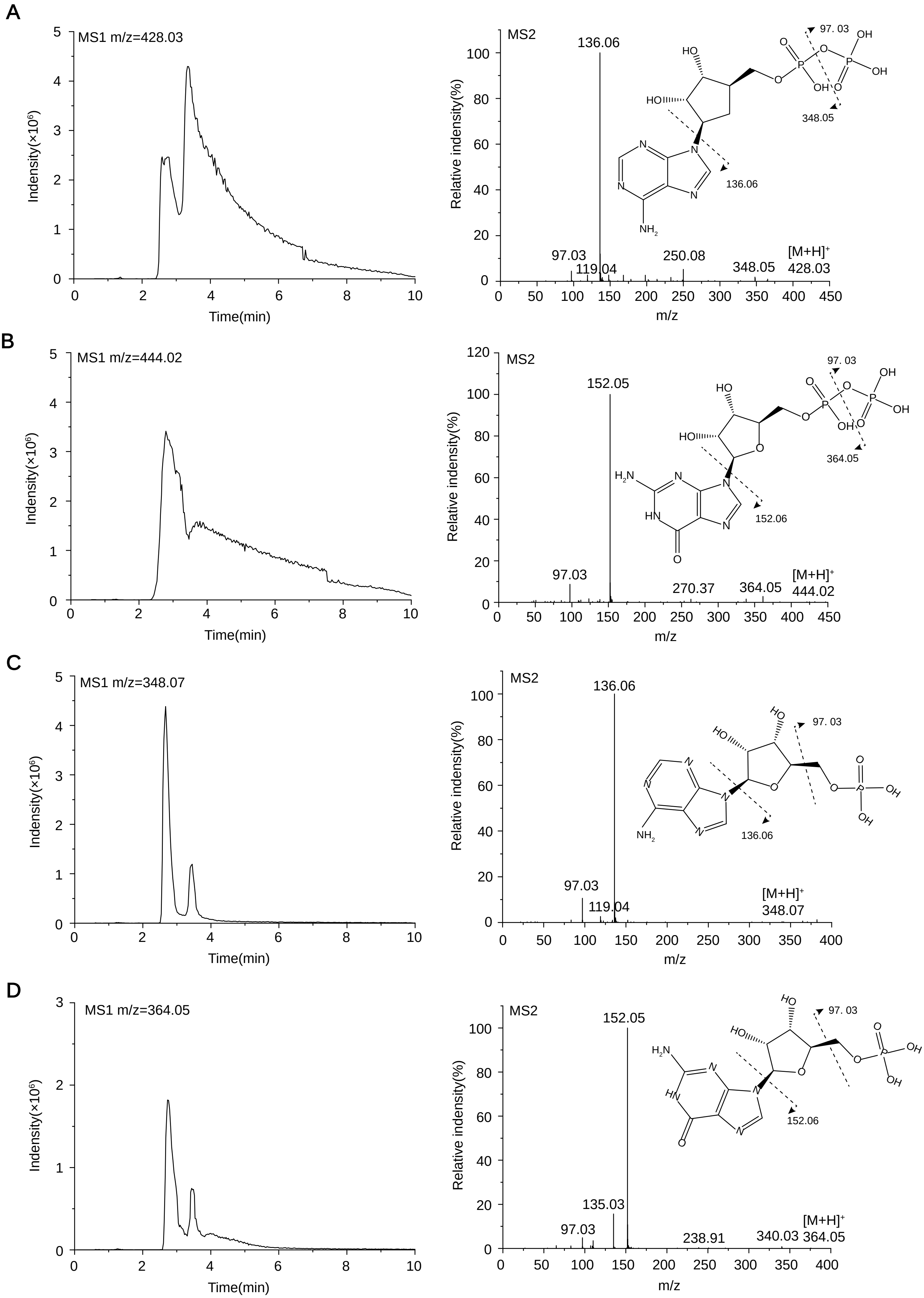

Figure S5. Mass spectrometric analysis of nucleotide. *A*, *B*, detection of reaction-derived ADP (*A*) or GDP (*B*) by LC-MS/MS. *C*, *D*, LC-MS/MS analysis of AMP (*C*) and GMP (*D*) generated in the reaction. Corresponding elution profile is shown in Fig. 3*B*.

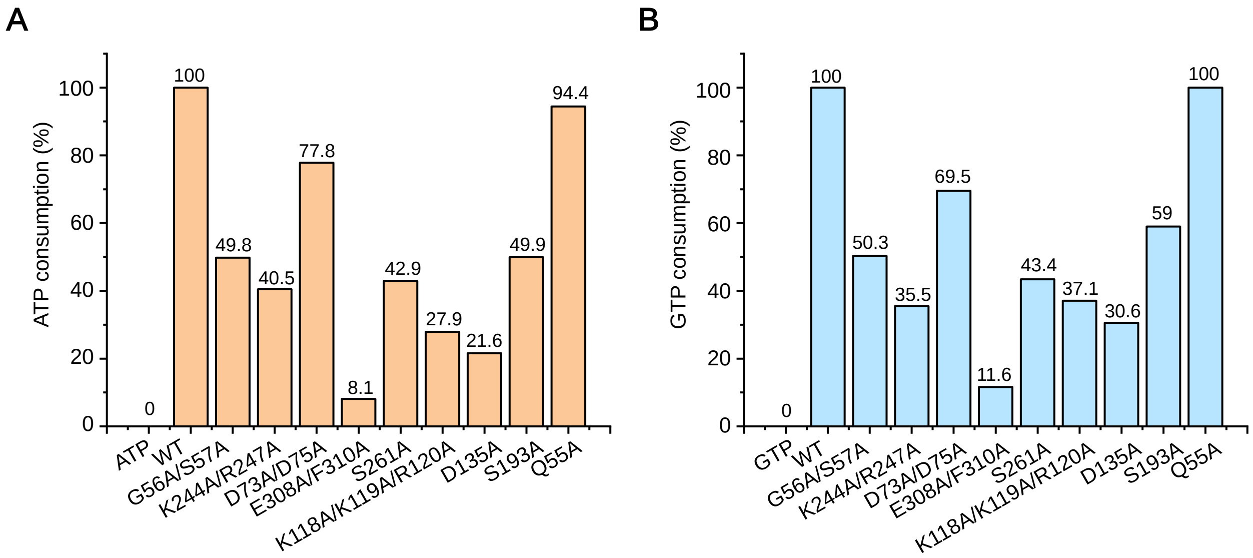

Figure S6. Quantitative analysis the catalytic activity of *Vc*CdnG. *A*, the substrate ATP consumption among diffe -rent mutants. *B*, the GTP consumption for each mutant.

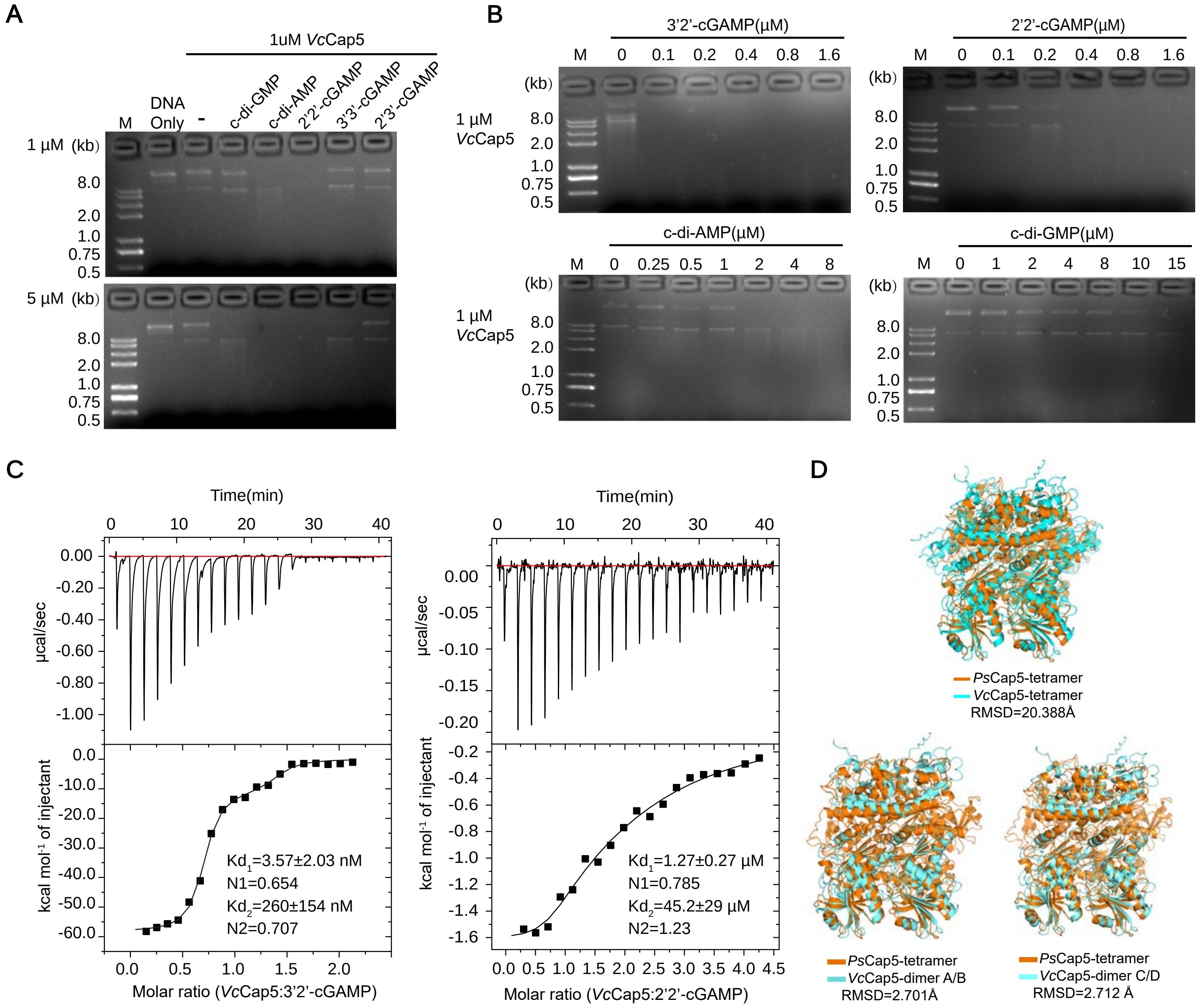

Figure S7. Functional characterization of *Vc*Cap5 activation by cyclic dinucleotides. *A*, second messenger-dependent activation of *Vc*Cap5 DNA cleavage. Nuclease activity assays were performed by incubating 1 µM *Vc*Cap5 with 10 ng/µl DNA in the presence of 1 µM or 5 µM of various cyclic dinucleotides. Each gel is a representative of at l east three independent experiments. *B*, dose-response analysis of *Vc*Cap5 activity with increasing concentrations of cyclic dinucleotides. The nuclease assay mixture contained 1 µM *Vc*Cap5, 10 ng/µl DNA, and cyclic dinucleotide over a concentration range of 0.1 to 15 µM. Each gel is a representative of at least three independent experiments. *C*, isothermal titration calorimetry (ITC) raw data (*top*) and binding isotherm (*bottom*) for the titration of 3'2'-cGAMP (*left*) and 2'2'-cGAMP (*right*) into *Vc*Cap5. *D*, structural superposition of the tetramer or dimer states, comparing the *Ps*Cap5 (PDB: 9NGL) and *Vc*Cap5. Each structural superposition panel displays the corresponding Cα RMSD value beneath it.

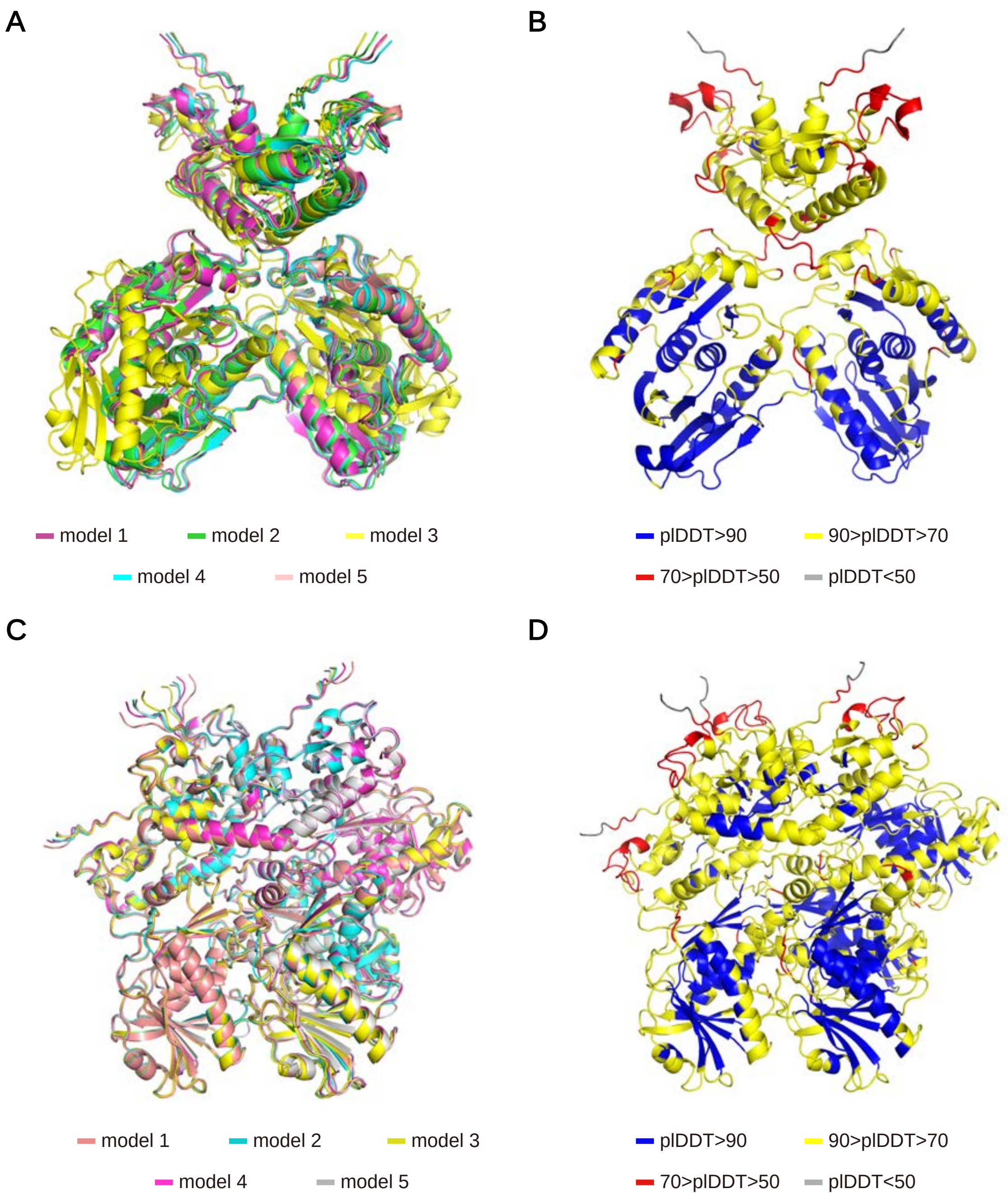

Figure S8. The *Vc*Cap5 dimer and tetramer structures computed by AlphaFold. *A*, superposition of the five best *Vc*CdnG dimer models. *B*, predicted structure of the *Vc*CdnG dimer colored by pIDDT: *blue* (pIDDT > 90), *yellow* (90 > pIDDT > 70), *red* (70 > pIDDT > 50), and *gray* (pIDDT < 50). *C*, superposition of the five best *Vc*CdnG tetramer models. *D*, predicted structure of the *Vc*CdnG tetramer colored by pIDDT: *blue* (pIDDT > 90), *yellow* (90 > pIDDT > 70), *red* (70 > pIDDT > 50), and *gray* (pIDDT < 50).
